# “Mitotic Slippage” and Extranuclear DNA in Cancer Chemoresistance: A Focus on Telomeres

**DOI:** 10.3390/ijms21082779

**Published:** 2020-04-16

**Authors:** Kristine Salmina, Agnieszka Bojko, Inna Inashkina, Karolina Staniak, Magdalena Dudkowska, Petar Podlesniy, Felikss Rumnieks, Ninel M Vainshelbaum, Dace Pjanova, Ewa Sikora, Jekaterina Erenpreisa

**Affiliations:** 1 Cancer Research Division, Latvian Biomedicine Research and Study Centre, LV-1067 Riga, Latvia; salmina.kristine@gmail.com (K.S.); inna@biomed.lu.lv (I.I.); felikss.rumnieks@gmail.com (F.R.); ninela.vainselbauma@biomed.lu.lv (N.M.V.); dace@biomed.lu.lv (D.P.); 2 Nencki Institute of Experimental Biology, Polish Academy of Sciences, 3 Pasteura St., 02-093 Warsaw, Poland; a.bojko@nencki.gov.pl (A.B.); k.staniak@nencki.edu.pl (K.S.); m.dudkowska@nencki.edu.pl (M.D.); e.sikora@nencki.edu.pl (E.S.); 3 CiberNed (Centro Investigacion Biomedica en Red Enfermedades Neurodegenerativas), IIBB, Rosello 161, 08036 Barcelona, Spain; ppodlesniy@gmail.com; 4 Faculty of Biology, University of Latvia, LV-1586 Riga, Latvia

**Keywords:** *mtTP53* cancer, genotoxic treatment, cellular senescence, polyploidization, extranuclear DNA, amoeboid conversion, ALT, inverted meiosis, budding of mitotic progeny, *SQSTM1/p62*

## Abstract

Mitotic slippage (MS), the incomplete mitosis that results in a doubled genome in interphase, is a typical response of *TP53*-mutant tumors resistant to genotoxic therapy. These polyploidized cells display premature senescence and sort the damaged DNA into the cytoplasm. In this study, we explored MS in the MDA-MB-231 cell line treated with doxorubicin (DOX). We found selective release into the cytoplasm of telomere fragments enriched in telomerase reverse transcriptase (hTERT), telomere capping protein TRF2, and DNA double-strand breaks marked by γH2AX, in association with ubiquitin-binding protein SQSTM1/p62. This occurs along with the alternative lengthening of telomeres (ALT) and DNA repair by homologous recombination (HR) in the nuclear promyelocytic leukemia (PML) bodies. The cells in repeated MS cycles activate meiotic genes and display holocentric chromosomes characteristic for inverted meiosis (IM). These giant cells acquire an amoeboid phenotype and finally bud the depolyploidized progeny, restarting the mitotic cycling. We suggest the reversible conversion of the telomerase-driven telomere maintenance into ALT coupled with IM at the sub-telomere breakage sites introduced by meiotic nuclease SPO11. All three MS mechanisms converging at telomeres recapitulate the amoeba-like agamic life-cycle, decreasing the mutagenic load and enabling the recovery of recombined, reduced progeny for return into the mitotic cycle.

## 1. Introduction

Once exposed to DNA damaging therapy, tumor cells (particularly *TP53* mutants) undergo a spindle checkpoint arrest, which can be released by mitotic slippage (MS), i.e. mitosis failure and reversal to interphase with a doubled genome [1]. Several laboratories independently found that, after passing several such polyploidizing cycles, a proportion of the surviving giant tumor cells undertake the reverse path, depolyploidization, returning “escapers” into the mitotic cycle [2,3,4,5,6,7,8]. Using different cancer treatment models, this recovery process was found to have several characteristic features: (a) duration of about one to three weeks; (b) tumor cell reprogramming [5,9]; (c) the death of most polyploid participants of the process leading to a remarkably small minority that inevitably survives severe DNA damage [2,9,10,11,12]; and (d) serves as a source of cancer metastatic relapse [13,14,15]. Although the amount of MS is roughly proportional to the drug dosage, it improves cancer cell survival [16].

The mechanisms of this MS-aided cancer resistance, which paradoxically integrates the features of cellular senescence with reprogramming, are poorly understood [8,17,18,19,20,21,22,23,24,25,26,27,28,29]. The paracrine tumor- and resistance-stimulating effects of the secretome of senescing cells are of interest [30] but the role of polyploidy as the third component of the paradoxical senescence–self-renewal duality of the chemoresistance is not sufficiently understood [8,26,31,32,33,34]. The release of extranuclear DNA in senescent cells via polyploidizing MS requires more study [10]. Extranuclear DNA was reported to be released in senescent cells through the defects or blebs in the nuclear lamina, and digested by lysosomal DNAse II, either directly or via macro-autophagy [35,36,37,38,39,40,41], causing Sting-mediated inflammation and suppression of innate immunity.

The capability of cancer cells to release cytosolic DNA enriched in DNA strand breaks in response to chemotherapy is proportional to the chromosome instability of cancer cell lines; surprisingly, this favors the epithelial–mesenchymal transition (EMT) and metastases in animal models [42]. MS and associated micronucleation may play a role in escaping cell death via sorting of the intrinsically damaged DNA [27]. However, the origin of this intrinsic damage, how sorting is regulated, and the cause of its survival advantage remain unanswered questions. A secondary origin of the DNA damage induced by chemotherapy and caused by upregulation of the meiotic program was proposed but only partly explored [12,43,44,45], leaving open the question of the mechanism and biological significance of the meiomitosis in cancer [46,47]. Here, we attempted to address these puzzles in the MDA-MB-231 cell line found previously to display a very high proportion of MS with cytosolic DNA [42]—by studying the response of this cancer cells line to the conventional chemotherapy drug doxorubicin (DOX), the inhibitor of topoisomerase II [48].

## 2. Results

### 2.1. Breast Cancer MDA-MB-231 Cell Line, before and after Doxorubicin (DOX) Treatment: The Phenotypes, Cell Growth, and Outlines of the Findings

**Figure 1:**
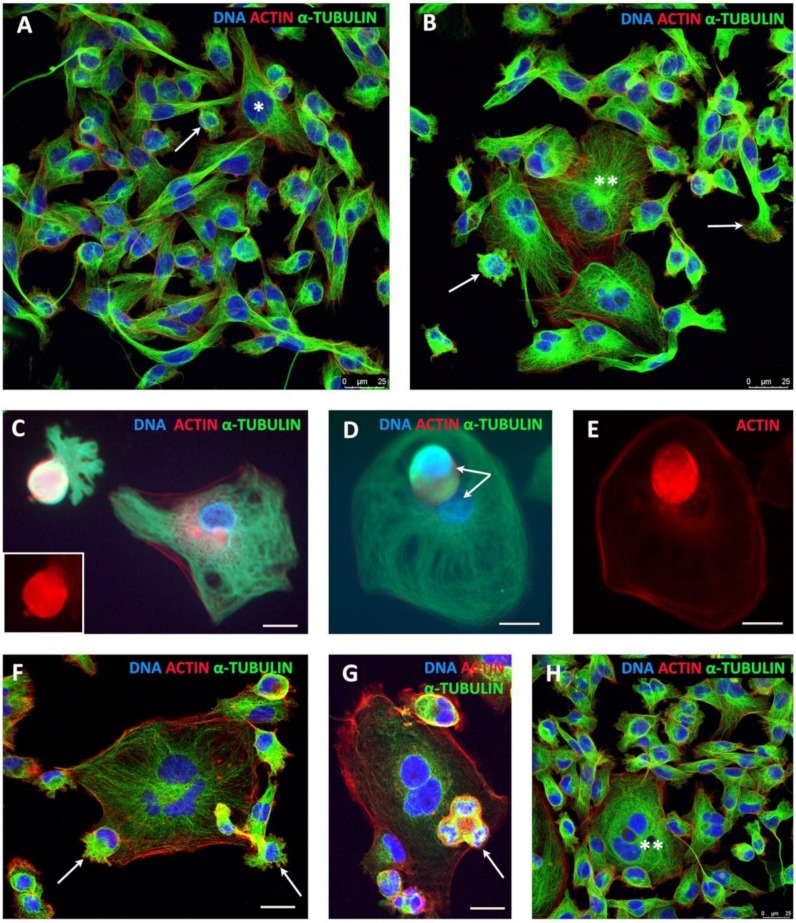
MDA-MB-231 cell culture (grown for 24 h in chamber slides), untreated and in the course of recovery after doxorubicin (DOX) treatment: (**A,B**) untreated control (arrows, mobile cells in epithelial–mesenchymal transition (EMT); * 8C; ** a multinuclear cell); (**C**–**E**) giant amoeboid cells on Day 13 post-DOX treatment budding spore-like subcells, which are extremely enriched in actin and tubulin; and (**F**–**H**) seven-week cell culture explanted from the escaped clone on Day 19 after DOX treatment. (**F**) A giant multinuclear cell is budding two subcells (arrows); the bi-polar ana-telophase on the right is spinning one daughter by the actin structure twisting around a spindle. (**G**) From the same culture, the progeny in a tripolar division (arrow) is situated on the giant cell. (**H**) View of the escaped clone with a general phenotype similar to the non-treated control. Bars = 25 µm.

**Figure 2:**
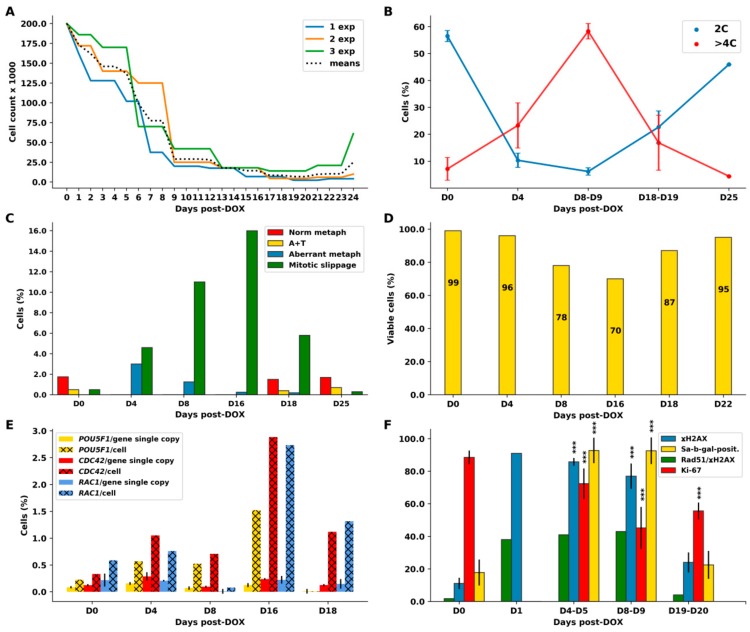
The quantified parameters of MDA-MB-231 cells following DOX treatment: (**A**) the cell growth curves of three independent experiments with a mean (a dashed line); (**B**) the reciprocal relationship between mitotic (2C) and polyploid (>4C) cell numbers overtime for three independent experiments (with SD); (**C**) representative differential mitotic counts (from Experiment 3, shown in (**A**) by the green line); (**D**) viability test with trypan blue from Experiment 3 (green curve in (**A**) and in (**C**)); (**E**) results of gene transcription evaluation obtained by Selfie digital PCR for three gene transcripts quantified per gene copy and per cell (as transcripts per gene copy multiplied by the average ploidy in the same experiment)—the average of three technical replicates with SEM; and (**F**) the dynamics of the senescence marker Sa-β-gal and proliferation marker Ki-67 along with DNA double-strand breaks (γH2AX) in three independent experiments and their repair by homologous recombination—cells with colocalized Rad51/γH2AX foci. (ANOVA with post hoc analysis (Tukey’s HSD test), *** *p* < 0.001.

This metastatic triple-negative breast cancer cell line was obtained from ECACC and cytogenetic analysis of its untreated culture was performed, confirming the reported characteristics [42]: a near-triploid karyotype with multiple chromosomal aberrations and karyotypic heterogeneity. MDA-MB-231 cell line is known to bear three oncogene driver mutations: *TP53R280K, KRASG13D,* and *BRAFG464V* [49]. In non-treated (NT) cell culture, it has a mostly fibroblastoid phenotype and contains a small proportion of polyploid cells (Figure 1A,B). After DOX treatment, the cells polyploidize, gradually acquire giant size, amoeboid phenotype, and by the end of the second week or later bud the mitotic progeny (Figure 1C–E) returning it to mitotic cycle (Figure 1F–H) and reconstituting the initial phenotype in escape clones (Figure 1H). During this process, the cell growth was seen steeply retarded in the second week and then very slowly elevated from the beginning of the third week (Figure 2A), when the first recovery clones appeared. The colony formation capacity was 0.009% ± 0.002% (*n* = 3). These are very small numbers. Despite this, in 16 experimental series performed on this model (each time seeing a very prolonged and significant drop in cell growth), the recovery consistently occurred. Trying to disclose the mechanisms of this incredible resistance, we studied several aspects of the recovery process—reversible polyploidy, reversible senescence, mitotic slippage, repair and sorting of the DNA damage, mechanisms of telomere maintenance, amoeboidization with the change of reproductive modus, and the involved genes—which all converged on telomeres and the atavistic variant of meiosis as a possible novel mechanism of survival escape. 

### 2.2. Cell Cycle Dynamics and Mitotic Slippage (MS) after DOX-Treatment

**Figure 3:**
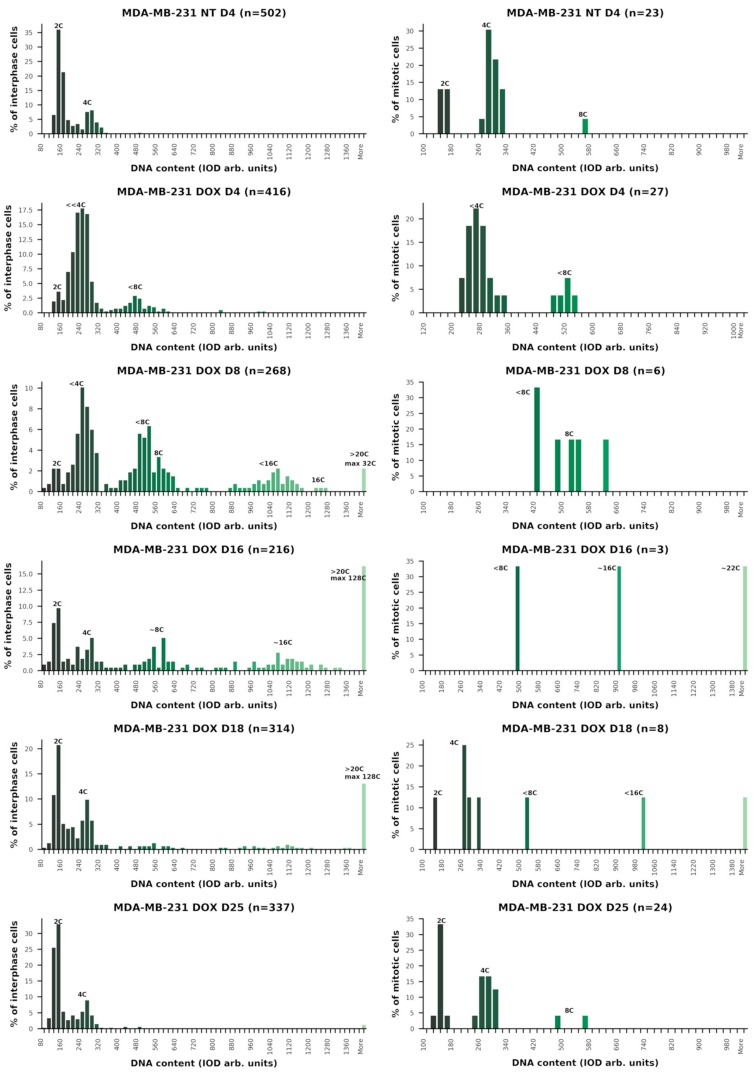
Representative cell cycle changes monitored with in situ DNA cytometry in the dynamics of the DOX treatment experiment: (**Left**) DNA histograms of interphase cell nuclei; and (**Right**) the DNA content in mitosis. Interruption of normal mitoses (Days 4–16) coincides with polyploidizing cycles. Bifurcation for depolyploidizing and continuing polyploidization sublines on Day 16 precedes resumption of the normal mitotic cycle on Day 18.

Cell cycle measurements by DNA cytometry revealed the induction of reversible polyploidy, in a reciprocal relationship with the mitotic cell cycle (Figure 2B, *n* = 3). A detailed description of the cell cycle changes, along with counts of aberrant mitosis, MS, and cell viability in one of the representative experiments (green line in Figure 2A) is shown in Figure 2C,D and the DNA histograms are presented in Figure 3. 

**Figure 4:**
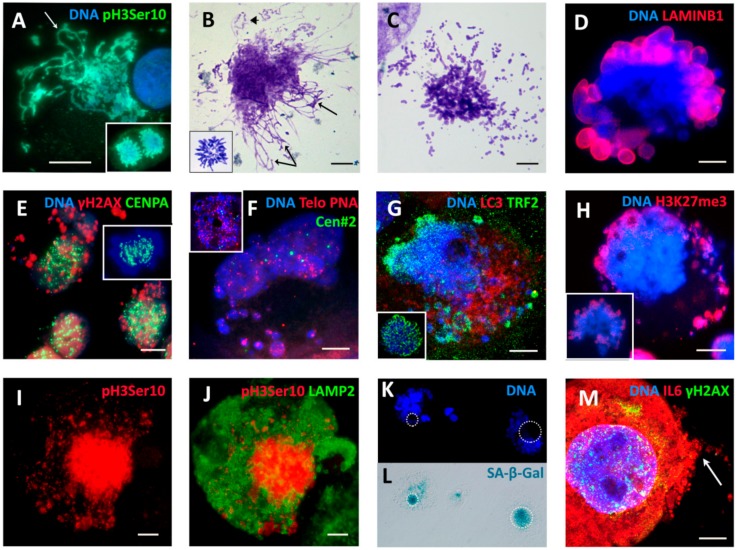
Characterization of aberrant mitosis and mitotic slippage (MS) by several in situ methods applied post-DOX treatment. Representative pictures from three or more experiments for each issue. (**A**–**C**) Aberrant metaphases with loopy chromosomes ((**A**,**B**) arrows) and their fragments, some circular ((**B**) arrowhead). (**A**) DOX-D4. (insert: normal anaphase of the non-treated (NT) control). (**B**,**C**) DNA staining with Toluidine blue (DOX-D16 and D18) (insert: normal 4C metaphase of NT). (**D**–**K**) Mitotic slippage: (**D**) defects of the lamina in the main nucleus and most cytoplasmic DNA clumps (DOX-D11); (**E**) holokinetic arrangement of kinetochores in the main nucleus and release of the damaged DNA into the cytoplasm (DOX-D5) (insert: metaphase of NT); (**F**) Fluorescence In Situ Hybridization (FISH) with the telomere and cen#2 probes showing retention of centromeres in the cell nucleus and release of a proportion of telomeres into the cytoplasm (DOX-D4) (insert: normal metaphase of NT); (**G**) preferential release of the telomere shelterin-TRF2-associated chromatin into the cytoplasm (DOX-D7) (insert: normal metaphase of NT); (**H**) the lamina-associated heterochromatin mark H3K27me3 (DOX-D5) showing partial release into the cytoplasm (insert: normal mitosis of NT with preferential localization of H3K27me3 mark on telomere ends); (**I**,**J**) early MS (yet pH3ser10-positive) with finely fragmented cytoplasmic DNA is surrounded by the LAMP2-positive lysosomal material (DOX-D7); (**K**,**L**) two cells in MS stained with 4′,6-diamidino-2-phenylindole (DAPI) for DNA and positively for Sa-β-gal (DOX-D4); and (**M**) a giant cell with senescence marks: DNA DSBs in cell nuclei and enrichment of cytoplasm with secreted (arrow) Interleukin-6 (IL6) (DOX-D8). Bars = 10 µm.

On Day 0, the non-treated (NT) cells had the usual DNA cytometry histograms for the cell cycle in interphase and for cell divisions (measured separately), with negligible admix of 8C cells (Figure 3, first row). The mitotic index was 2.2% (Figure 2C), divisions proceeded in a bi-polar manner as shown in Figure 4A (insert) and, in rare cases, also through tripolar mitosis. Besides major 4C, the 8C metaphases were also encountered (Figure 3, first row, right).

On Day 4 (one day of 100 nM DOX treatment plus three days after the change of the medium), as shown in Figure 3 (second row), the majority of cells accumulated in late S–G2-phase with strongly under-replicated DNA (<<4C), 3% of cells were arrested in metaphase (Figure 2C), whereas a subpopulation of the under-replicated cells had already overcome the barrier of tetraploidy, doubling its DNA content, reaching a peak <8C, and also accumulating in the next <8C metaphase arrest. The arrested metaphases displayed aberrant features, without exception (Figure 2C), and contained DNA double-strand breaks (DSB), as detected by γH2AX (not shown). Microscopy performed on Days 4–5 revealed the disordered metaphase figures, often with loopy chromosomes lacking sister cohesion, and the peripheral linear or circular chromosome fragments. Their typical appearance (also characteristic for further days after DOX-treatment) is shown in Figure 4A–C. Anaphases and telophases on Days 4–5 were absent. A proportion of the arrested metaphases undergo MS—a reversal to interphase with the doubled genome (for the counts, see Figure 2C). The microscopic patterns of MS are shown in Figure 4D–K. MS is usually exhibited by one large interphase nucleus with irregular contours and LAMIN B1 defects, surrounded by chromatin clusters and lamin-enveloped micronuclei (Figure 4D). Clusters of the chromatin released into the cytoplasm are enriched with DNA double-strand breaks (DSBs), which were densely stained for γH2AX (Figure 4E).

On Day 8 (Figure 3, the third row), the cells further underwent the genome duplication cycles, forming ~4C, ~8C, and ~16C peaks of under-replicated DNA, and, in small proportions, the euploid 8C and 16C peaks. A small amount of ~32C cells also appeared. The degree of the DNA under-replication, compared by DNA cytometry on Day 4, diminished (Figure 3) and the proportion of aberrant metaphases also dropped; they were near-octoploid, but normal cytokinetic cell division still had not resumed (Figure 2C). Reciprocal to the decrease in the proportion of aberrant metaphases, the proportion of MS increased along with the degree of polyploidy (Figure 2C and Figure 3), thus these cells more easily reset interphase after spindle checkpoint adaptation. All cells undergoing mitotic slippage continued to sort the increasing amounts of damaged DNA into the cytoplasm. Simultaneously, many cells detached from the flask bottom and died. As a result, by Days 7–9, the cell number steeply decreased (Figure 2A), however, about 80% of the cells attached to support excluded Trypan blue, i.e. were viable (Figure 2D).

On Day 16, a dramatic change occurred: the proportion of cells in the polyploidization cycles revealed on the DNA cytometry histograms as ~4C, ~8C, and ~16C peaks diminished, while the proportion of diploid cells increased (Figure 3, the fourth row, left). The back-route of this part of the DNA histogram points to the start of the depolyploidization phase for recovery. However, cytokinetic mitoses were not found (they could be extremely rare); only very aberrant polyploid metaphases with continued chromosome looping and fragmentation of the peripheral chromatin were observed (Figure 3, the fourth row, right); one is depicted in Figure 4B. The proportion of hyperploid cells (>32C) and mitotic slippage continued to increase (to 16% in both counts; Figure 2C). This bifurcation in the DNA histogram points toward the separation of the two giant cell sublines: one undergoing depolyploidization starting with ~32C–16C cells and the other continuing to re-replicate DNA and undergo MS. Thus, the proportion of mitotic slippage on Days 8 and 16 continued to increase, while 4C mitoses and 2C anaphases were still not evident.

The time point of bifurcation of the two cell lines coincided with the minimum of the cell growth curve (Figure 2A) and the lowest viability (70%) of the cells attached to support (Figure 2D), both signaling the population crisis. At this critical point, we recorded the highest average DNA content per cell (12.15 C; Table 1, first column). Some cells reached 128 C-ploidy. It means that giant and supergiant cells were dominating, filling up the cell population by their mass. In some other experiments with somewhat slower recovery, the maximum encountered DNA amount in the (endo)metaphase was 52C and the largest interphase cell reached 396 C DNA content (not shown).

On Day 18, the normal cell cycle was restored (Figure 3, the fifth row, left) and normal cytokinetic mitoses (with 2C anaphase/telophase) reappeared (Figure 3, the fifth row, right). The proportion of hyperploids and MS reciprocally decreased (Figure 2C and Figure 3); however, a small amount of the polyploidizing cycles and MS (continuing to sort the damage-signaling DNA) was still encountered for several weeks. The cell number started to increase (Figure 2A, green line). In the other two experiments presented in Figure 2A (blue and orange lines), this regrowth started a few days later.

On Days 24–25 (Figure 3, the sixth row), the cell cycling was similar to the NT control with a larger proportion of ana-telophase/metaphase (Figure 2C), pointing to hasty cell divisions. Mitotic slippage became negligible (<0.2%), as in the NT control (Figure 2C). However, rare giant cells were still present in the samples.

A summary of the typical relationship between observed cell cycle phenomena presented in Figure 2A–C and Figure 3 in their dynamics after DOX treatment is provided in Table 1.

In Table 1, the chain of events can be deduced as follows: DOX causes DNA under-replication, M-arrest, and MS, initiating polyploidization cycles, which increase ploidy through further rounds of re-replication coupled with repeated MS. However, between the second and third weeks (in the particularly described experiment, it was recorded on Day 16), the two diverging polyploid sublines emerged, one starting depolyploidization and the other continuing the increase in ploidy through repeated cycles of re-replication and MS for a few more days until the stabilization of the normal cell cycle. When this was achieved, hyperploid cells gradually died (as observed by necrosis).

This inevitable survival outcome recovering a very small proportion of resistant cells poses a question of how it is possible. This is the main question of cancer resistance to therapy. Several phenomena with unusual features accompany the process of cancer cell recovery after DOX treatment through the reversible polyploidy. The most striking is the budding of mitotic progeny from late giant cells; the phenomenon has been previously described [3,5,6,50,51,52,53] and termed “neosis” [3]. We found that it is part of the amoeboid conversion of giant cells. Below, we provide a short description of our model.

### 2.3. The Amoeboid Transition of Polyploidized Breast Cancer Cells and Budding of the Mitotic Progeny

By the end of the second week after DOX treatment onward, most cells reached a diameter of 50–300 µm, acquired a powerful microtubular cytoskeleton, enriched with actin, also nuclear (Figure 1C–G), and sometimes encysted (Figure S1A). On chamber slides, we observed the giant cells assuming variable shapes (Figure 1C and Figure S1D, and not shown), some with front–rear polarity, pseudopodia, lamellipodia, and filopodia, which indicate that the EMT-amoeboid invasion phenotype was acquired [54]. Some polynuclear giant cells began to bud spore-like cellularized progeny, highly enriched with microtubules and actin (Figure 1C–E and Figure S1B,D). The budded progeny usually immediately performed bi-polar (more rarely tripolar or tetrapolar) cell division, sometimes even before or during the process of budding (Figure 1F,G) or after homing at the surface of their “mothers” or the nearest giant neighbor (Figure S1C and not shown). The remaining nucleus of a giant cell was often seen in the process of autophagy induction (Figure S1B) and deterioration (Figure S1C). At this very early stage of recovery, the new-born small and giant cells seemed to be cooperating as a system, with the giants providing not only nutritive but also mechanical support for the young generation (Figure S1C,D). Rare budding from polynuclear giant cells was still found even 7–10 weeks post-DOX treatment (Figure 1F); however, on the whole, the culture phenotype returned to that of the untreated cells (Figure 1H). We decided to check the cells for the transcription of the Rho GTPases that are responsible for cell motility involved in the amoeboid transition, cell membrane protrusion for spore budding, and for invasion, *CDC42* and *RAC1* [55,56], as well as for the main stemness transcription factor *POU5F1/OCT4A* in the prolonged time course post-DOX. The results of Selfie digital PCR expressed per gene copy and per cell (multiplied by the average ploidy number, shown in Table 1, determined in the same culture) for each sampling term are presented in Figure 2E, per gene copy and gene dosage. These genes were upregulated on Day 4 (starting with the ploidy cycles); by Day 16, the three markers were particularly high due to high gene dosage in giant cells. Later, together with the recovery of the normal mitotic cycle and reciprocal decrease of polyploidy, the amoeboid regulators went down. Thus, amoeboid phenotype was linked to the DOX-induced polyploidy, and through the spore-like intermediates triggered the recovery of escape mitotic clones. As mentioned above, during the entire prolonged transition period, up to recovery of the mitotic cell cycle, cell polyploidization was accompanied by mitotic slippage, releasing cytoplasmic DNA, and this phenomenon was further studied.

### 2.4. Extranuclear DNA Released by MS in DOX-Treated Cells Contains Telomere Heterochromatin Enriched with the TRF2-Shelterin but Not Centromeres

**Figure 5:**
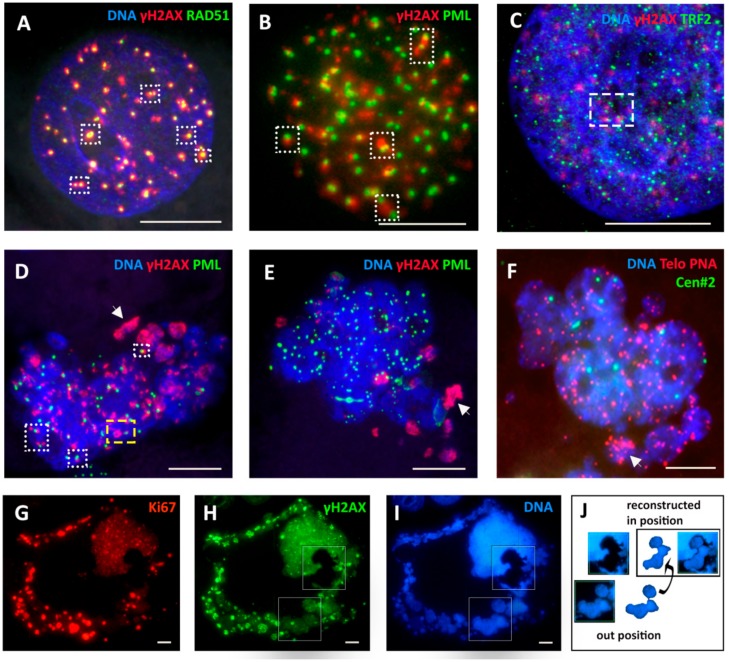
Representative pictures of DNA repair of telomere DNA double-strand breaks by RAD51-dependent homologous recombination (HR) involving promyelocytic leukemia (PML) bodies, a sign of alternative lengthening of telomeres (ALT) in giant post-DOX cell nuclei undergoing MS cycles with sorting the damage signaling DNA out into the cytoplasm and reconstituting subnuclei free of it: (**A**–**D**) Days 5–8 post-DOX (*n* = 3). The typical HR configurations are boxed, the extranuclear damaged DNA on (D) arrowed. (**E**,**F**) Reconstitution of subnuclei in two similar cells (DOX-D8-9): (**E**) the extranuclear damaged DNA (arrowed) does not contain PML bodies; and (**F**) FISH with the telomere and cen#2 probes (*n* = 3) showing the telomere label cluster in the extranuclear DNA (arrowed). (**G**–**I**) The release of four repaired subnuclei (boxed) from a defect in the giant mother nucleus (reconstructed in **J**); high Ki-67 positivity of the sorted DNA signaling damage by γH2AX-label on DOX-D19 (*n* = 3). Bars = 10 µm.

The aberrant arrested metaphases contain DNA double-strand breaks (Figure 4B,C); however, during MS, a considerable amount of the fragmented chromatin is sorted into the cytoplasm, thus the remaining nucleus becomes free of the DNA damage (Figure 4E). This creation of the extranuclear damaged DNA and its sorting do not decrease; they increase with repeated cycles of polyploidization and the accompanied MS. To elucidate if there is any cytogenetic selectivity of the sorted DNA, we stained cell nuclei for centromeric kinetochore protein CENPA, telomere capping shelterin TRF2 [57,58], and performed Fluorescent in Situ Hybridization (FISH) with the telomere-sequence-specific probe combined with the probe for chromosome 2 centromere, for internal control. The typical results of both experiments on Days 4–9 post-DOX-treatment are presented in Figure 4E–G and Figure 5F. We found that in MS the centromeres/kinetochores remain within the restituted cell nuclei. The telomeres in the MS nucleus as compared with NT control (on insert) have variable size and the tendency to cluster; part of the telomere sequenced label was observed in cytoplasmic clusters of the damage-signaling chromatin. The extranuclear DNA was enriched with telomere-capping protein TRF2 (Figure 4G). We also paid attention to the fact that, in arrested metaphases undergoing MS and extending loopy chromosomes into the cytoplasm, their ends are often closed and circular structures detach (Figure 4A,B). The multiple kinetochores were arranged along the chromosomes of the MS nucleus in tandem arrays (Figure 4E), while the centromeres of chr#2 were normally represented by three doublets in control metaphase (Figure 4F, insert) and six separate dots in a (4C) slippage nucleus (Figure 4F). DNA methylation determined by the antibody to 5′-methyl-cytosine after DOX revealed the decreased methylation in the lamin-associated marginal chromatin as well as poor DNA methylation in some clusters of extranuclear chromatin in MS. The H3K27me3-marked heterochromatin, which in control metaphases is located at telomere ends (Figure 4H, insert), becomes partially released from cell nuclei into cytoplasmic clumps in MS (Figure 4H), apparently along with LAMIN B1 defects (compare Figure 4D,H). The intensity of the lysosome marker LAMP2 was enhanced by DOX before the mitosis-slipping cells became deprived of the mitotic marker pH3ser10 staining (Figure 4I,J). In other words, the damage-signaling DNA that is released from the cell nucleus during MS is enriched with the fragmented and circularized telomere heterochromatin but is selectively void of centromeres and kinetochores which are likely indispensable for repeated cycles of MS. The chromosomes in aberrant metaphases and MS, however, exhibited holokinetic distribution of kinetochores, weak or absent sister chromatid cohesion, and often closed ends. Simultaneously, these same cells display cellular senescence.

### 2.5. The Features of Cellular Senescence in the MDA-MB-231-DOX-Treated Cells with Persistent DNA Damage, Ki-67 Positivity, and HR DNA Repair are Related to MS

In our MDA-MB-231-DOX model, we registered the typical hallmarks of cellular senescence: a high percentage of Sa-β-gal-positivity (Figure 2F), including MS cells (Figure 4K,L), persistent DNA damage (Figure 2F) associated with the Il6-rich secretome (Figure 4M) [49], LAMIN B1 insufficiency (Figure 4D), and release of extranuclear circular DNA (Figure 4B and see below). These canonic senescence hallmarks were combined with the opposite features— positive cell cycle hallmark Ki-67 and DNA repair by homologous recombination (HR)—in a large proportion of these cells (Figure 2F). The p21 senescence marker was also upregulated by DOX [49] but the terminal senescence CDNK4A/p16 protein was usually observed sequestered in cytoplasmic vacuoles. Both groups of opposite phenomena were upregulated by DOX and expressed in the same polyploid cells (at least, half of them) over the period of two weeks (Figure 2F) but became downregulated with restitution of the mitotic cycle, also in accordance. Our data suggest that restitution of the mitotic progeny from these polyploid amoeboid cells seen as budding (Figure 1C–F) was prepared during this prolonged senescence period including the repeated MS. Erosion of telomeres accompanies cellular senescence and favors a transition to tetraploidy [59,60]. The cytogenetic study and staining for TRF2 showed that telomere marks were observed both in restituted giant nuclei and in the released chromatin. The question then became: How are telomeres involved in the DNA repair and sorting of cytoplasmic DNA during MS?

### 2.6. Homologous Recombination (HR) Repair in Giant Interphase and MS Cells is Associated with Promyelocytic Leukemia (PML) Bodies and is Featured by Alternative Lengthening of Telomeres (ALT)

The aberrant metaphases that emerged in the first cell cycles after DOX were all positive for DNA double-strand breaks. From the γH2AX-positive cell counts, presented in Figure 2F, about 85% of interphase cells possessed the DSBs in the first week, and nearly as many in the second week after DOX. Similar counts showed the proportion of SA-β-gal-positive cells and about half of them were involved in HR (Figure 2F). This persistent DNA damage signaling and persistent HR continued through the transient period of polyploidization and amoeboid conversion. Applying a detailed microscopic study of HR during Days 5–10 post-DOX (three independent experiments), besides colocalization of RAD51 and γH2AX foci in giant cells (Figure 5A), we unexpectedly found another participant of the DSB repair process—the PML nuclear bodies (Figure 5B,D)—a marker of alternative telomere lengthening (ALT). ALT-associated PML bodies (APBs) are known to provide the nuclear compartment for the ALT mechanism, clustering and aligning the telomere ends together, for the homologous recombination repair of the eroded telomere on the non-defective double strand [61].

The APB bodies join telomeres for HR with the invading RAD51 (with or without RAD52) filament, so PML bodies become colocalized with RAD51 foci [61]. In post-DOX polyploid interphase cells and in the body of some restituted nuclei of MS cells, we found these typical compositions of the relationship between the foci of the doublets of the γH2AX-positive chromosome fragments and RAD51 single and double foci, and the same for PML (boxes in Figure 5A,B,D). The foci of telomere shelterin TRF2 were also found in a similar relationship with γH2AX fragments in some giant cell nuclear parts (boxed in Figure 5C). This mechanism was likely involved in the sorting of the damaged DNA, as we found PML within the repairing giant cell nuclei and MS associated with foci of γH2AX and Rad51. However, PML bodies were absent in the γH2AX-labeled DNA cytoplasmic clumps (Figure 5D,E). Here, we present three MS cells in the deduced dynamics of the repair process. HR DNA repair and sorting are occurring in the cell MS nucleus in Figure 5D (DOX-Day 5) where the typical configurations are boxed. We see the formation of a circular non-repaired DNA, still linked to PML, at the nuclear border (yellow box), which might then be released into the cytoplasm. Clusters of the damaged DNA are separated in the cytoplasm (arrowhead). Figure 5E shows MS in the development of the process (DOX-Day 10), with the restituted giant nucleus free of the DNA damage, reconstructing into subnuclei, each with its own nucleolus, while clusters of the damaged DNA are separated in the cytoplasm (arrowhead). Figure 5F shows a very similar composition in the FISH sample (DOX-Day 9), where two reconstituting subnuclei contain 2 + 4 cen#2 labels and multiple telomere labels, while the discarded chromatin is enriched with the clustered telomere label (arrowhead). The next row (Figure 5G–J) shows a giant cell (DOX-Day 19) performing the nuclear release of four repaired sub-nuclei (boxed in Figure 5H,I and reconstructed in Figure 5J) with simultaneous sorting of large amounts of the damaged DNA (γH2AX-positive). The whole-cell DNA content measured by DAPI was about 32C. The main nucleus and its four extruded sub-nuclei were free from DNA damage signaling and weakly stained for Ki-67. The huge amount of the damaged discarded chromatin was colocalized with bright Ki-67 staining (positive in MS also at earlier terms), which may suggest the amplification of this material with the rolling circle mechanism. Indeed, this cytoplasmic DNA material shows the unscheduled DNA synthesis in BrdU pulse experiments (Figure S2A,B). 

### 2.7. Telomere Ends are Released into the Cytoplasm by MS

**Figure 6:**
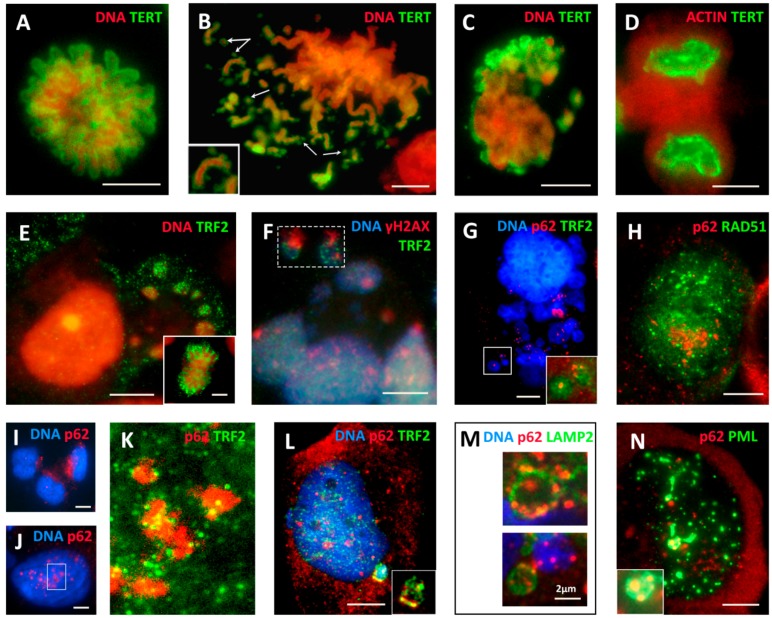
(**A**–**F**) The release of the TERT-TRF2-γH2AX-marked chromatin into the cytoplasm of DOX-treated cells (DOX-Days 5–8, if not specified otherwise; *n* ≥ 3) accompanied by the autophagy adaptor SQSTM1/p62: (**A**) TERT-positive metaphase in NT control (DNA counterstained by propidium iodide); (**B**) separation of the TERT-enriched circular fragments (arrows) in the restituting polyploid metaphase (insert: a chromosome doublet ending by two circular structures); (**C**) mitotic slippage with TERT-enriched cytoplasmic DNA, poor in the nucleus (DOX-Day 5); (**D**) the conventional TERT-positivity in the escape telophase cell (DOX-Day 22); (**E**) a giant cell after MS surrounded by clumps of extranuclear DNA enriched with TRF2 (insert: the TRF2-positive telophase of the NT); (**F**) a fragment of the giant MS cell with two boxed chromatin TRF2-enriched fragments marked by DNA damage (γH2AX) in the cytoplasm; (**G**) the giant MS cell with abundant extranuclear chromatin enriched with TRF2 and attached SQSTM1/62 protein using RGB filter (the green channel was removed) (insert: from the boxed fragment, the blue channel is removed); (**H**) the bi-nuclear giant cell with RAD51 repair foci that are apart from the cluster of p62 foci; (**I**) p62 is scarce in the nuclei of NT cells; (J) nuclear clustering of p62 foci in a giant DOX-treated cell; (**K**) enlarged nuclear fragment boxed in (**J**) with removed blue DAPI channel reveals the attachment of TRF2 foci to the p62 clusters; (**L**) an octoploid cell with several p62 nuclear foci surrounded by TRF2 and a cytoplasmic chromatin fragment, shown enlarged in the insert without the blue channel with clear colocalization of TRF2 and p62; (**M**) cytoplasmic fragments of the MS cell co-stained for p62, LAMP2, and DNA showing accumulation of p62 in the membrane of lysosomal vesicles, perhaps attracting cytoplasmic DNA to them; and (**N**) the giant cell in the terminal senescence with typical nuclear polymorphic enlarged PML bodies accumulating p62 (enlarged on the insert). Bars = 10 µm.

To study the process further, we applied the antibody for the catalytic unit of telomerase, reverse transcriptase (hTERT), and combined the available markers of the ALT process (PML, RAD51, and γH2AX) with TRF2 immunofluorescent staining. We conducted a detailed microscopic study, focusing on the nuclear and cytoplasmic distribution of the components in MS. The results are presented in Figure 6A–F.

MDA-MB-231 cells are known for maintaining their telomeres by telomerase and this cell line is strongly positive for hTERT in the non-treated control outlining the metaphase chromosomes (Figure 6A). During the post-DOX transition of the aberrant mitosis into repeated MS, multiple circular hTERT-positive DNA structures were observed being released into the cytoplasm (Figure 6B), resulting in the relative enrichment of discarded cytoplasmic DNA fragments with hTERT (Figure 6C); this process was also observed in some late giant cells on Day 19 (Figure S2C). However, in the recovery clones, the hTERT staining is again in its place (Figure 6D). The monoclonal antibody for TRF2, similarly, stains normal telophases of the untreated control (Figure 6E, insert) but enriches the released cytoplasmic DNA after DOX treatment (Figure 4G and Figure 6E). This material contains the γH2AX-positive fragments as presented in Figure 6F (boxed). Thus, these data show that, during MS, the DNA structures of the telomere-end origin, which are often circularized, are transferred into the cytoplasm, signaling DNA damage. From these observations, we suggest that, in giant MS cell nuclei, ALT is substituting the telomerase-driven repair of telomeres, while the telomerase overhang and rolling circles are cut off into the cytoplasm retaining hTERT. In turn, having been repaired by ALT-HR, the trimmed telomeres in cell nuclei interrupt the DNA damage signaling and further bud the mitotic progeny shifted back to the telomerase maintenance of telomeres. 

These observations explain the two-week-long coupling between DNA DSBs and HR repair documented in Figure 2F. However, the source of the secondary DNA DSBs for this intrusive coupling with HR discarding the chromosome ends in giant cells is unclear. The most immediate source of DSBs is the under-replication of heterochromatin caused by DOX-induced topoisomerase II inhibition, leading to accumulation of DNA DSBs [62] associated with fragile sub-telomeric sites of the late-replicating heterochromatin [63]. However, over time, the under-replication induced by DOX decreases (Figure 3) but MS, with the release of extranuclear DNA, increases along with continued nuclear HR up to recovery of the mitotic cycle by Days 18–20, when both decrease (Figure 2C,F).

The recombination repair of telomeres by ALT needs the whole-genome homology search of the complementary telomere matrices for the eroded ones. Arnoult and Karlseder [64] revealed the rapid whole-nuclei spanning of telomeres involved in ALT and suggested that ALT participates in whole-genome homology search by a mechanism evolutionarily related to meiosis. Amoeboid sporogenesis-like mechanism of reproduction also puts the question about possible meiosis. Therefore, further, we focused on the expression of meiotic features and meiotic genes in our model.

### 2.8. The Meiotic Features of “Mitotic Slippage”

**Figure 7:**
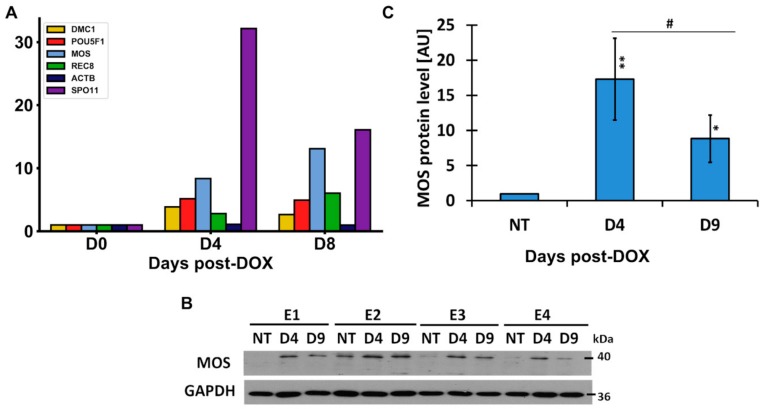
Expression of the meiotic genes and proteins after DOX treatment. (**A**) RT-PCR results of gene transcription are shown in folds. Representative charts of two independent experiments, with three technical replicates; (**B**) MOS protein induction on Days 4 and 9 after DOX treatment, analyzed by Western Blot in four independent experiments. (**C**) The protein level of MOS; densitometry analysis of Western blot bands (*n* = 4) (the *p*-value is stated as: * 0.01 < *p* < 0.05; ** 0.001 < *p* < 0.01); # statistical significance between subsequent days of DOX treatment.

In the untreated control, this cell line expressed very low meiotic *MOS-kinase* but the transcription was several-fold enhanced after DOX-treatment (Figure 7A). Western blot analysis also revealed the upregulation of MOS after DOX treatment (Figure 7B,C).

**Figure 8:**
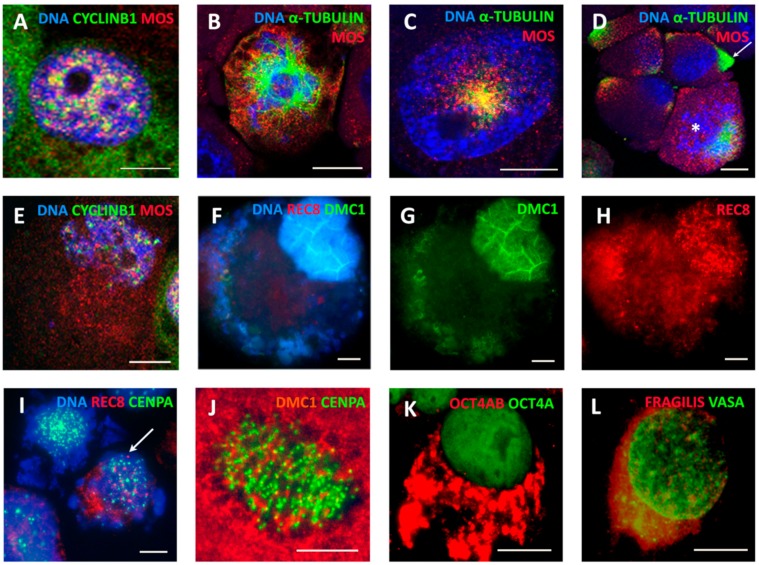
Expression of meiotic and germline proteins in MS and giant cells found by Immunofluorescence—representative images for at least three experiments: (**A**) a tetraploid cell nucleus enriched with MOS-kinase (sc-86) colocalized and juxtaposed with CYCLIN B1 (DOX-D2); (**B**) an attachment of MOS to the centrosomes and microtubules of the tripolar mitosis (DOX-D4); (**C**) MOS and α-TUBULIN form a monopolar spindle in the early prophase (DOX-D4); (**D**) MOS is attached to interphase centrosomes (arrow) and shows a remnant of a monopolar spindle in MS (asterisk) [12]; (**E**) the restituting nucleus in MS becomes poor with MOS and CYCLIN B1 (DOX-D4); (**F**–**H**) a giant cell in MS releasing cytoplasmic DNA shows the enrichment of the cell nucleus with DMC1 (meiotic recombinase) and REC8 (meiotic cohesin) (DOX-D19); (**I**) REC8 grains are scarcely inserted in the kinetochore chains in the MS cell (arrow) (DOX-D4); (**J**) DMC1 grains are scarcely inserted in the MS cell (DOX-D7; [12]); (**K**) a giant cell enriched with OCT4A in the cell nucleus (a monoclonal Ab) and OCT4B in the cytoplasm (DOX-D5); and (**L**) a giant cell enriched with the germ markers, DDX4/VASA in the cell nucleus and FRAGILIS in the cytoplasm (DOX-D7). Bars = 10 µm.

In the control, MOS-staining was found by immunofluorescence (IF) being colocalized or juxtaposed with cyclin B1 in rare G2 cell nuclei and in some metaphases, but this expression was enhanced in DOX-treated cells from the second day post-DOX treatment (Figure 8A). MOS-protein was also seen interacting with a centrosome in interphase cells, spindle poles in arrested metaphases (Figure 8B,C), and forming a monopolar spindle (needed for the homology search) in the prophase-like cells (Figure 8C, detailed in [12]). A remnant monopolar spindle was occasionally found in some MS cells (Figure 8D, asterisk), which usually contained two centrosomes (not shown). In the development of MS, MOS, and CYCLIN B1 proteins degraded along with the restitution of the interphase nucleus (Figure 8E). 

In general, the IF observations can be interpreted as imposing by MOS-kinase of the meiotic prophase upon mitosis, likely starting between the G2 and M arrest. The notable changes in MOS production coincide with the period of the emergence of aberrant “metaphases”, MS, and release of extranuclear chromatin (Figure 2C and Figure 4A). However, even stronger upregulation from practically zero levels in the control was found in the transcription of *SPO11* meiotic nuclease induced by DOX (Figure 7A, DNA contamination excluded). Alongside, we found mild transcription increase of the meiotic cohesin *REC8* and meiotic recombinase *DMC1* after DOX treatment (Figure 7A). These proteins were, however, scarcely inserted in the tandem chains of kinetochores in some arrested and slipping metaphases (Figure 8I,J) but accumulated in the MS nuclei of later giant cells (Figure 8F–H; DOX-Day 19, comparable with Figure 5G–I). These data allowed us to link MS and the release of extranuclear DNA enriched with telomere sequences with a meiotic component (more detailed mechanistic studies on this aspect are planned). The DOX-treated giant cells (heterogeneously) acquired higher positivity for OCT4A/POU5F1 (also confirmed by RT-PCR (Figure 2E and Figure 7A), whereas its alternatively spliced variant OCT4B also can be very strong in the cytoplasm (Figure 8K) and the truncated *OCT4B1* variant, which was expressed in the control, considerably increased. The giant cells were also positive for other germline markers (DDX4/VASA protein, found also in non-treated cells), but the marker of the oocyte maturation, FRAGILIS, was found particularly enhanced by DOX in giant cells (Figure 8L). As already indicated, the holocentric chromosomes in DOX-treated MS cells typical for inverted meiosis (IM) were also identified (Figure 4E and Figure 8J) (more details and control are described in [12]). Thus, it was revealed that the repeated cycles of MS possessed in many aspects the meiotic features. 

### 2.9. Extranuclear Sorting of the Cut-Off Telomere Ends in MS Being Regulated with the Participation of the Polyubiquitination Adaptor Protein SQSTM1/p62

The question remains if cell degrading systems are involved in the extranuclear sorting of cut-off telomere ends. SQSTM1/p62, a multifunctional polyubiquitination adaptor protein that plays an important role in cell signaling and autophagic degradation of protein aggregates, may be involved. We noted that the damaged chromatin released into the cytoplasm was also enriched with p62 foci (Figure 6G), but, in the repairing nuclei, the p62 clusters (if present) avoided colocalization with RAD51 foci (Figure 6H) but were surrounded by attached and embedded TRF2 foci (see Figure 6I,K,L for NT control), and colocalized with TRF2 in the extranuclear chromatin fragments (Figure 6L, boxed and shown without DAPI in insert). The microscopy using staining for DNA, lysosome activating protein LAMP2, and p62 did not rule out the mediator role of p62 for the link of the damaged telomere DNA to lysosomes (Figure 4I and Figure 6M). However, in terminally senescing cells, where PML bodies enlarge and become polymorphic, p62 was poorly exported from cell nuclei but became incorporated into these enlarged nuclear PML bodies (Figure 6N). These observations correspond well with the data reported by Hewitt et al. [65] on the role of the HR-negative regulating function of p62 in ALT by degradation recombination proteins, in particular RAD51. From this study, we also conclude that the release of the circular damaged DNA into the cytoplasm is part of the ALT telomere repair process processed by HR in the cell nucleus and influenced by p62. In agreement with the opinion of Hewitt et al. [65], this mechanism for genome integrity surveillance very likely depends on the autophagic flux, whose capacity in terminally senescing cells can become exhausted, leading to excessive accumulation of p62 [20]. A more detailed investigation of the degradation pathways of the soluble DNA was outside the scope of the current study.

## 3. Discussion

After treatment of breast cancer MDA-MB-231 cells with DOX, we observed several phenomena: (1) cellular senescence; (2) polyploidization by mitotic slippage; (3) activation of meiotic genes; (4) extranuclear sorting of the cut-off circularized telomere ends along with the signs of the telomere maintenance mechanism by ALT in these polyploidized cell nuclei; and (5) amoeboid conversion of these cells with final sporogenesis-like budding of their depolyploidized progeny restarting mitotic cycling. In terms of cell mobility, the process of epithelial-to-mesenchymal-amoeboid transition is associated with metastatic cancer spread [54]. All these complex phenomena need to be placed into a mutual context explaining, in particular, why MS improves cell survival [16].

The dynamics of these processes that we evaluated over the period from the mitotic cycle of the untreated population up to its return in survivors, two to three weeks after DOX treatment, may provide a clue. Firstly, the relationships between mitotic and polyploidy cycles are clearly reciprocal. Secondly, the shift from telomerase-driven to ALT-like TMM was also reversible. Thirdly, the amoeboid conversion associated with polyploidy cycles through MS and the change of the reproduction modus, from mitotic to sporogony-like, was also reversible. All these concerted changes induced by 24 h 100 nM DOX treatment continued for 10–14 days in the background of the drug-induced cell senescence, which hence was also not terminal. We speculate that the transient shift in TMM was associated with the change of the tumor cell reproduction modus serving a prerequisite for its realization. The amoeboid cell conversion, including reproduction by spores, is akin to the asexual lifecycle of unicellular organisms [52,53,66,67]. Therefore, these coordinated events could not be random but bore the features of the atavistic program executed in quite a prolonged time.

One of the central observations in this model was the change of the TMM in the MDA MB 231 cell line, known as telomerase-driven, for ALT. The well-known markers of ALT include a high level of telomere–sister chromatid exchanges (may be seen as closed chromosome ends); extrachromosomal circular telomere repeats; and a specialized telomeric nuclear structure, ALT-associated PML [68]—all these hallmarks were found in our MDA MB 231-DOX model. Analysis of the literature showed that this change of TMM in the telomerase-driven tumors is possible, and is favored by cellular senescence and telomeric DNA damage [68,69,70] and we observed both. Although the telomerase mechanism is characteristic for epithelial cancers, while primary ALT for the mesenchymal ones [70], the epithelial tumor cells with depleted hTERT undergo epithelial–mesenchymal transition (EMT) and ALT [71]. Again, we observed this transition to ALT after physical depletion of hTERT with its substrate (in the cytoplasm) of amoeboid cells. In turn, stable expression of PML in telomerase-primed MCF7 breast cancer cells also results in ALT [72], whereas ectopic expression of hTERT in primary ALT cells maintains their telomeres with both TMMs [68,69]. The control of telomere length by trimming of telomere ends, generating the amplifying circles, was reported as likely involving the ALT mechanism [73], which is considered by us here, too.

However, a source of these telomere trimming breaks and the biological advantage of the TMM shift for survival escape remained elusive. Here, we found that the process occurs along with upregulation of meiotic genes along with self-renewal and germ master gene *OCT4A* and some other germ genes—*VASA* and *FRAGILIS*. The expression of meiotic genes in cancers with poor prognosis and their involvement in resistance to irradiation and drugs are known and have been studied [12,43,44,45,74,75,76,77,78,79,80]. However, the mechanisms of so-called “meiomitosis”, whose evolutionary origin was earlier proposed [81], are poorly understood, and whether they stabilize or destabilize the genome is currently disputed [46,47,76,82].

**Figure 9:**
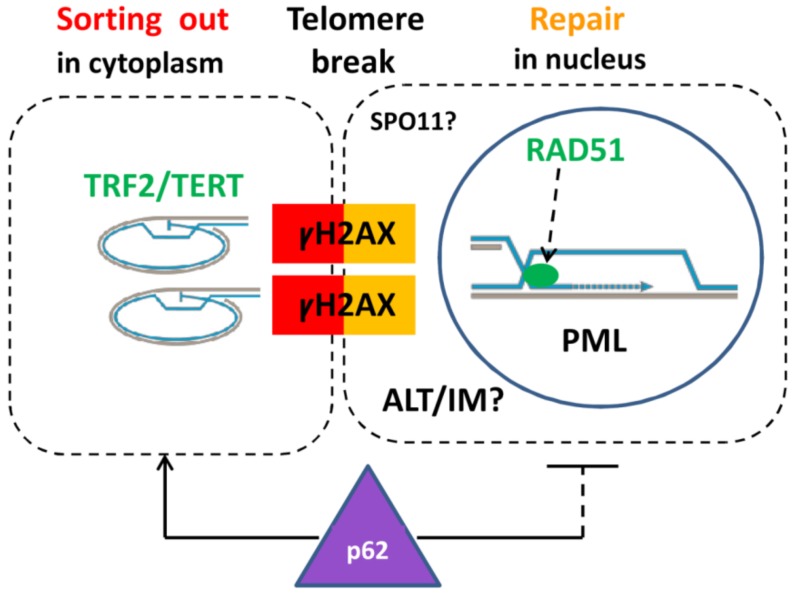
A schematic showing the cytoplasmic sorting of hTERT/TRF2-marked DNA damage signaling telomere ends cut off by a telomere break during mitotic slippage. This process is associated with the ALT-RAD51-driven repair by homologous recombination of the two co-aligned trimmed telomeres occurring in specific nuclear PML (APB) bodies. ALT may be coupled with inverted meiosis (IM) by recombining homologous chromosomes conjugated by telomeres at the same breakage site. In this case, the breakage sites can be introduced by meiotic nuclease SPO11. The ubiquitination protein SQSTM1/62 participates in the sorting of the extranuclear DNA.

The observed MOS-kinase associated phenotypes suggested the imposing of the meiotic prophase onto the cells in G2M arrest caused by DOX. Among the cohort of upregulated meiotic genes in our model, we found the meiotic recombination nuclease *SPO11*. The SPO11 nuclease is a conservative regulator known from archaea [83] that can introduce the DNA double-strand breaks in the telomere fragile sites [84]. We suggest here for the first time that ALT may be coupled by HR in polyploid tumor cells with inverted meiosis (IM) in PML-APB bodies for recombination (and possibly counting) of homologous chromosomes. If so, SPO11 may drive the whole process of TMM shift during MS by cutting off the telomere ends, as schematized in Figure 9, and possibly sorting the non-homologous chromosome pairs. 

The IM, which has only recently received wider attention from researchers, is an evolutionary ancient variant of meiosis in which the homologous chromosomes specifically synapse and undergo recombination by their sub-telomeric ends, terminalize chiasma [85], and segregate sisters in the first and homologs in the second meiotic division [86,87,88,89,90]. For that, holocentric (holokinetic) chromosomes with diffuse kinetochores distributed along the chromosome length, capable of karyokinesis but disabling the centromeric cohesion of sister chromatids, are needed. We found holocentric chromosomes in DOX-treated MDA MB 231 cells undergoing polyploidization and MS and recently described some more elements of IM in this and other human tumors, such as irradiated Burkitt’s lymphoma [12]. IM has been reported not only in protists, plants, and insects but also in each third normal human oocyte, sorting the non-recombined chromosomes in aneuploid polar bodies [91,92]. Thus, whereas ALT in human tumors is compatible with the telomerase mechanism of TMM [68,93], the IM is compatible with monocentric chromosomes, in both human tumors and embryos. In addition, TERT was shown stabilizing telomere caps even without telomere lengthening and reversibly leaving cell nuclei under stress conditions [94]. 

IM coupling to telomere maintenance by ALT (or even substituting it) could resolve the long-standing puzzle of the Muller’s Ratchet [95] in the obligately agamic amoeba existing on Earth for eons. They undergo cyclic polyploidy accompanied by chromatin diminution and express the orthologs of genes employed in meiosis of sexual eukaryotes *Spo11, Mre11, Rad50, Rad51, Rad52, Mnd1, Dmc1, Msh,* and *Mlh* [83,87,96,97,98,99]. Archetti [100] recently presented calculations showing that asexual reproduction can replace sexual reproduction with inverted meiosis due to recombinative gene conversion, providing protection from the deleterious loss of heterozygosity and outweighing the cost of sex. The same consequence and biological advantage can enable the use of polyploidy and IM as an archaic adaptive mechanism that limits hypermutation, for tumor cell immortality and resistance to extinction.

The nutritive significance of soluble cytoplasmic DNA and the mechanical support of the budded cancer “spores”, which we observed in this study homed and likely fed with the nucleotide soup by the giant amoeboid “mothers and aunts” before they were able to stabilize clones and the microenvironment on their own (Figure S1B,C), should not be underestimated. The divergence of the reproductive depolyploidizing fraction and the apparently vegetative “nurses”, continuing to re-replicate and produce soluble DNA through MS until stabilization of the normal mitotic cycle, all acting as a tissue system, is an example of the histiotrophic co-operation, which was also noted by Zybina and Zybina [101,102] in the development of the mammalian trophoblast.

Finally, we briefly discuss the relationship of the phenomenology in DOX-treated breast cancer cells to cell senescence. We observed six hallmarks of premature cell senescence: Sa-β-gal-positivity, persistent DNA double-strand breaks [103], reduction of lamin B1, the release of heterochromatin [35,36], erosion of telomeres associated with tetraploidy [60], and secretion of cytokines (in particular, IL6) during a prolonged period post-DOX damage [49]. However, we simultaneously found that the persistent chromatin damage and its repeated sorting from cell nuclei occurring during polyploidization cycles are necessarily coupled with proliferation, self-renewal, and meiotic markers, and bear a programmed characteristic discussed above. The dependence of reprogramming capacity on accelerated senescence is clearly established [8,24,25,32,104]. Thus, although displaying basic hallmarks of senescence, for quite a long period of time, the DOX-treated polyploidizing MDA-MB-231 cells are not really senescent but rather reproductive (paradoxically, both).

In conclusion, the present results investigating the link between cellular senescence, MS, and the processes of ALT and IM in chemoresistant cancer cells, all converging on telomeres, open a new avenue for further research and possible targeting.

## 4. Materials and Methods 

### 4.1. Cell Line and Treatment

The breast adenocarcinoma MDA-MB-231 cell line was obtained from the ECACC (European Collection of Authentic Cell Cultures, Wiltshire, UK). Cells were cultured in flasks in Dulbecco’s modified Eagle’s media (DMEM) supplemented with 10% fetal bovine serum (FBS; Sigma-Aldrich, St. Louis, MO, USA) at 37 °C in a 5% CO_2_ humidified incubator without antibiotics. For experimental studies, cells were maintained in the log phase of growth and treated with 100 nM DOX (doxorubicin) for 24 h. After drug removal, cells were maintained by replenishing culture medium every 2–3 days and sampled over a 3-week period post-treatment until the appearance of escape clones. In some, we further followed the re-seeded recovery fractions for 5–8 weeks. Some issues were repeatedly addressed in the shorter time periods, provided that the recovery of escape clones was further achieved. In some experiments, cells were grown on chamber slides. 

For testing the unscheduled DNA replication, the cells were incubated with 5 μM BrdU for 90 min before sampling.

### 4.2. Cell Growth, Viability, and Colony Formation 

To determine growth kinetics, cells were seeded and treated at a density of 200,000 cells per well in a 6-well plate and counts were performed using a Neubauer camera (Heinz Herenz Medizinalbedarf GmbH Hamburg, Germany) and Trypan blue dye (0.4%) exclusion. After treatment, cell number changes were monitored at several time points until the recovery. Cell viability was calculated as the proportion of the cells attached to the support with Trypan blue exclusion.

To determine colony formation capability, 1.5–2 million cells in a T25 flask were treated with 100 nM DOX. On Day 22 after treatment, the cells were rinsed with phosphate-buffered saline (PBS), then fixed and stained with Trypan blue, and the number of colonies exceeding 50 cells was counted. Colony formation capability was calculated as a percentage of the initial cell number before treatment with DOX. 

### 4.3. Immunofluorescence

Cells were pelleted, suspended in warm FBS, and pelleted by cytocentrifuge again onto polylysine-coated glass slides or grown and fixed on chamber slides. Cytospins were fixed in methanol for 7 min at –20 °C, dipped 10 times in ice-cold acetone, and allowed to briefly dry. Slides were then washed three times in Tris-buffered saline (TBS) 0.01% Tween20 (TBST) for 5 min. Slides were subsequently blocked for 15 min in TBS, 0.05% Tween 20%, and 1% bovine serum albumin (BSA) at room temperature. Samples were covered with TBS, 0.025% Tween 20%, and 1% BSA containing the primary antibody, and incubated overnight at 4 °C in a humidified chamber. Samples were then washed three times in TBST and covered with TBST containing the appropriate secondary antibodies (goat anti-mouse IgG Alexa Fluor 488, goat anti-rabbit IgG Alexa Fluor 594, or sheep anti-human IgG Alexa Fluor 488 (Invitrogen, Carlsbad, CA, USA)) and incubated for 40 min at room temperature in the dark. Slides were washed three times for 5 min with TBST and once for 2 min in PBS. Samples were then counterstained with DAPI (0.25 μg/mL) for 2 min or with propidium iodide (5 µg/mL) and finally embedded in Prolong Gold (Invitrogen, Carlsbad, CA, USA). When staining for α-tubulin, actin, or IL-6, the post-fixation drying step was omitted and fixation in 4% paraformaldehyde was performed. For 5-methylcytosine and BrdU staining, DNA denaturation was performed by 2 N HCl at 37 °C for 20 min before the blocking step. Primary antibodies and their sources are listed in Table 2.

For microscopic observations, a fluorescence light microscope (Leitz Ergolux L03-10, Leica, Wetzlar, Germany) equipped with a color video camera (Sony DXC 390P, Sony, Tokyo, Japan) and laser scanning confocal microscope (LEICA TCS SP8, Wetzlar, Germany) were used. To capture fluorescent images, in addition to separate optical filters, a three-band BRG (blue, red, green) optical filter (Leica, Wetzlar, Germany) was used.

### 4.4. Toluidine Blue DNA Staining and Image Cytometry

Cytospins were prepared and fixed in ethanol/acetone (1:1) for >30 min at 4 °C and air-dried. Slides were then hydrolyzed with 5 N HCl for 20 min at room temperature. In some experiments, shortened hydrolysis (30–60 s) was performed, preserving cytoplasmic RNA staining. Slides were then washed in distilled water (5 × 1 min) and stained for 10 min with 0.05% toluidine blue in 50% citrate-phosphate McIlvain buffer at pH 4. Slides were rinsed with distilled water, blotted dry, and dehydrated by incubating twice in butanol for 3 min each at 37 °C. Samples were then incubated twice in xylene for 3 min each at room temperature before being embedded in DPX. Digital images were collected using a Sony DXC 390P color video camera (Sony, Tokyo, Japan) calibrated in the green channel. DNA content was measured as the integral optical density (IOD), using Image-Pro Plus 4.1 software (Media Cybernetics, Rockville, MD, USA). The stoichiometry of DNA staining was verified using the values obtained for metaphases compared with anaphases and telophases (ratio 2.0); arbitrary diploid (2C) DNA values were averaged from measuring anaphases in untreated tumor cells. The method error was estimated to be less than 10%. For cell cycle measurements, 200–500 interphase cells were collected at each point. For DNA measurements, 500–2000 cells were collected for each point. The counts of the normal and aberrant mitoses and mitotic slippage were recorded microscopically per 500 or more cells.

### 4.5. Fluorescence in Situ Hybridization (FISH) 

Cells were harvested, washed with warm PBS, treated with 75 mM KCl at room temperature, incubated for 40 min at 37 °C, and centrifuged. Serum and KCl solution (1:1) were added to cells and suspension was pelleted by cytocentrifuge on slides and air-dried. Then, slides were fixed for 1 h with two changes of fresh methanol/glacial acetic acid (3:1) at –20 °C. Telomere FISH for Telo PNA Cy3/Cen#2 FITC was done with a peptide nucleic acid (PNA) telomere probe (Dako Inc., Glostrup, Denmark) in conjunction with a differentially colored centromere 2 PNA probe (a gift from Dako, Inc., Glostrup, Denmark) as an internal reference point as previously described [105,106]. FISH was carried out using 1 M NaSC pretreatment (56 °C for 20 min), denaturation step for 3 min at 83 °C and hybridization at 37 °C overnight. Denaturation and hybridization steps were performed on a ThermoBrite (Leica, Wetzlar, Germany) programmable temperature controlled slide processing system. Slides were mounted in antifade solution (Vector Laboratories, Burlingame, CA, USA) or in Prolong Gold with DAPI (Invitrogen, Carlsbad, CA, USA).

### 4.6. Detection of Senescence-Associated-β-Galactosidase

Detection of senescence-associated-β-galactosidase (SA-β-gal) was performed according to [107]. Briefly, cells were fixed in 2% formaldehyde and 0.2% glutaraldehyde in PBS, washed, and incubated overnight at 37 °C with a solution containing 1 mg/mL 5-bromo-4-chloro-3-indolyl-β-d-galactopyranoside, 2.5 mM potassium ferrocyanide, 2.5 mM potassium ferricyanide, 150 mM NaCl, 2 mM MgCl_2_, and 0.1 M phosphate buffer, pH 6. Cell nuclei were stained with DAPI (1 μM in PBS) or Hoechst 33342 (1 μg/mL in PBS). Cells were observed under a Nikon Eclipse Ti (Nikon, Tokyo, Japan), a fluorescent microscope with a 40×/0.6 Nikon lens, and analyzed using the NIS Elements Basic Research software. The percentage of SA-β-gal positive cells was calculated.

### 4.7. RT-PCR

Total RNA was extracted from MDA-MB-231 (10^6^ cells) using TRIZOL (Invitrogen, Carlsbad, CA, USA). First-strand cDNA was synthesized using 2 µg of RNA, random hexamers, and RevertAid^TM^ M-MuLV Reverse Transcriptase (Fermentas, Vilnius, Lithuania) according to the manufacturer’s instructions, and subsequently diluted with nuclease-free water 10 times. The absence of contamination with chromosomal DNA was verified by PCR using ®-actin primers (Table 3). Amplification was carried out in a total volume of 20 μL with 2 μL of diluted cDNA, 4 μL of 5× HOT FIREPol^®^ Blend Master Mix (Solis BioDyne, Tartu, Estonia) and the following primers: *OCT4A* F/R, *OCT4B* F/R, *OCT4B1* F/R, *MOS* F/R, *REC8* F/R, *DMC1* F/R, and *SPO11* F/R under the manufacturer’s recommended conditions. Amplified PCR products were analyzed by direct sequencing after ExoI/FastAP treatment (Thermo Scientific, Waltham, MA, USA) using the fluorescent Bigdye Terminator v3.1 Cycle Sequencing protocol on a 3130xl Genetic Analyzer (Applied Biosystems, Waltham, MA, USA) and verified using the BLAST NCBI (National Center for Biotechnology Information) platform (Bethesda, MD, USA). Amplified PCR products were visualized on 1.2% agarose gels and quantified using ImageJ software [108].

### 4.8. Selfie-Digital RT-PCR

To preserve the content of the nucleic acids in the sample, selfie-digital PCR analysis was performed using lysate as previously described [111]. Briefly, cell culture media were aspirated from the culture flasks and the cell layer was lysed in 100ST DNA/RNA/Protein Solubilization Reagent (#DCQ100ST, DireCtQuant, Lleida, Spain) at 250,000 cells/mL. The lysate was incubated at 90 °C for 3 min, centrifuged at 10,000 rpm for 10 min and the supernatant used directly. Strand-specific, absolute expression level analysis of gene transcription was performed using Selfie-digital PCR as previously described [111]. The reverse transcription step was performed using the reverse primer listed in Table 4. Digital PCR was performed using EvaGreen SuperMix (186-4005, Bio-Rad, Hercules, CA, USA) on the QX200 ddPCR platform. PCR amplification was performed in a thermal cycler (C1000 Touch Thermal Cycler, Bio-Rad, Hercules, CA, USA) using the following thermal profile: 95 °C 5 min; 40 cycles of 95 °C for 30 s and 60 °C for 1 min; 4 °C for 5 min; and 90 °C for 10 min. Non-template controls containing all the reagents and the corresponding amount of solubilization buffer without sample lysate were included in all steps of the procedure. The number of RNA transcripts was calculated by subtracting the number of amplicons measured in the reaction without reverse transcriptase (RT-) from the reaction with reverse transcriptase (RT+) and dividing by (RT-). The results are expressed as the number of transcripts per gene. The specificity of the primers was checked using BLAST analysis and the correct size and homogeneity of the amplicons after gel analysis of the PCR products.

### 4.9. Western Blot Analysis

Living adherent cells were harvested into Laemmli SDS sample lysis buffer, sonicated, and centrifuged at 10,000× *g* for 10 min. The concentration of proteins was estimated by the BCA method; 100 mM DTT and 0.01% bromophenol were added to lysates before separation by SDS-PAGE (12% gels were used). Total protein concentrations were determined using bicinchoninic acid (BCA) protein assay kit, according to the manufacturer’s instructions. The same protein amount (20 μg) was loaded into each well. Membranes were blocked in 5% nonfat milk dissolved in TBS containing 0.1% Tween-20 for 1 h at room temperature (RT). Then, the membranes were probed overnight at 4 °C with primary antibodies. The respective proteins were detected after incubation with the horseradish peroxidase-conjugated secondary antibodies (1:2000) (Dako, Glostrup, Denmark), using an ECL system (Thermo Scientific, Rockford, IL, USA; according to the manufacturer’s instructions).

### 4.10. Statistical Analysis 

Statistical analysis was performed with the use of the STATISTICA 11 program, TIBCO Software Inc, Palo Alto CA, USA. ANOVA (analysis of variance) was used for the analysis of differences among three or more groups, followed by post hoc analysis (Tukey’s honestly significant difference; HSD test). Normal distribution of the data was tested with the Shapiro–Wilk test. The *p*-value was stated as: * 0.01 < *p* < 0.05; ** 0.001 < *p* < 0.01 *** *p* < 0.001.

## Figures and Tables

**Table 1:** The dynamics of the cell cycle and MS after DOX treatment.

Sample	Average Ploidy *	Normal Cycle	Aberrant Metaphase	Mitotic Slippage	Polyploidy Cycles	Hyperploidy > 20C	Normal Mitosis
Day 0	2.68C	+	−	−	−	−	+
Day 4	3.65C	− ^1^	++	+	+	−	−
Day 8	7.47C	−	+	++	+++	+	−
Day 16	12.15C	+/−	+/−	+++	++	+++	-
Day 18	8.87C	+	−	+	+	++	+
Day 25	3.26C	+	−	−	−	−/+	++

* For the experiment presented on Figure 3; ^1^ severe DNA under-replication.

**Table 2:** The antibodies used, their specificity, and source.

Antibody Against	Description	Specificity/Immunogen	Used Concentration	Product No. and Manufacturer
AURORA B	Rabbit polyclonal	A peptide derived from within residues 1–100 of human Aurora B	1:300	ab2254, Abcam, Cambrige, UK
BrdU	Mouse monoclonal	Recognizes the thymidine analog 5-bromo-2′-deoxyuridine (BrdU)	1:100	A21300, Invitrogen, Carlsbad, CA, USA
α-Tubulin	Mouse monoclonal	Recognizes an epitope located at the C-terminal end of the α-tubulin isoform in a variety of organisms	1:1000	T5168, Sigma-Aldrich, St. Louis, MO, USA
Centromere protein	Human	Derived from human CREST patient serum	1:50	15–234, Antibodies Inc., Davis, CA, USA
CYCLIN B1	Mouse monoclonal	Raised against a recombinant protein corresponding to human cyclin B1	1:100	sc-245, Santa Cruz, Dallas, TX, USA
DMC1	Mouse monoclonal	Specific for DMC1, does not cross-react with the related protein Rad51	1:100	ab11054, Abcam, Cambridge, UK
F-ACTIN		Phalloidin-iFlour 594 Conjugate	1:500	ab176757, Abcam, Cambridge, UK
FRAGILIS	Rabbit polyclonal	The details of the immunogen for this antibody are not available	1:50	ab65183-100, Abcam, Cambridge, UK
GAPDH	Mouse monoclonal	Raised against recombinant GAPDH of human origin	1:50000	sc-47724, Santa Cruz, Dallas, TX, USA
IL6	Rabbit polyclonal	Synthetic peptide	1:50	orb87798, Biorbyt, Cambridge, UK
γ-H2AX	Rabbit polyclonal	Recognizes mammalian, *yeast Drosophila melanogaster* and *Xenopus laevis* γ-H2AX	1:200	4411-PC-100, Trevigen, Gaithersburg, MD, USA
γ-H2AX	Mouse monoclonal	Synthetic peptide sequence surrounding phosphorylated Ser140	1:200	Ma1-2022, Pierce, Waltham, MA, USA
H3K27me3	Rabbit polyclonal	Synthetic peptide within human Histone H3 aa 1–100	1:200	ab6147, Abcam, Cambridge, UK
Ki67	Rabbit polyclonal	Synthetic peptide from C-terminus of human Ki-67	1:50	PA5-16785, Pierce, Waltham, MA, USA
LAMIN B1	Rabbit polyclonal	Peptide mapping at the C-terminus of Lamin B1 of human origin	1:200	ab1604, Abcam, Cambridge, UK
LAMP2	Mouse monoclonal	The details of the immunogen for this antibody are not available	1:500	555803, BD Pharmingen™, Franklin Lakes, NJ, USA
MOS (C237)	Rabbit polyclonal	Epitope mapping at the C-terminus	1:50	sc-86, Santa Cruz, Dallas, TX, USA
MOS	Rabbit polyclonal	Synthetic peptide corresponding to a region within internal sequence amino acids 107–156	1:500	Ab99017, Abcam, Cambridge, UK
OCT 3/4	Mouse monoclonal	Peptide raised against amino acids 1–134 of Oct-3/4 of human origin non-cross-reactive with Oct-3/4 isoforms B and B1	1:50	sc-5279, Santa Cruz, Dallas, TX, USA
OCT4	Rabbit polyclonal	A peptide derived from within residues 300 to the C-terminus of human Oct4	1:200	ab19857, Abcam, Cambridge, UK
*p*-AMPKα1/2 (Thr183/172)	Rabbit polyclonal	Epitope corresponding to phosphorylated Thr172 of AMPKα1 of human origin	1:50	sc-101630, Santa Cruz, Dallas, TX, USA
pH3Ser10	Mouse monoclonal	Recognizes Phospho- S10 on Histone H3	1:200	ab14955, Abcam, Cambridge, UK
PML	Mouse monoclonal	Epitope corresponding to amino acids 37–51 mapping near the N-terminal of PML of human origin	1:200	sc-966, Santa Cruz, Dallas, TX, USA
P62/SQSTM1	Rabbit polyclonal	A synthetic peptide corresponding to Human SQSTM1/ p62 (C-terminal)	1:200	ab91526, Abcam, Cambridge, UK
RAD51	Mouse monoclonal	Recombinant full-length protein corresponding to human Rad51 aa 1–338	1:50	ab213, Abcam, Cambridge, UK
REC8	Rabbit polyclonal	Peptide mapping near the N-terminus of Rec8 of human origin	1:50	10793-1-AP, Proteintech Group, Manchester, UK
TERT	Mouse monoclonal	Recombinant full-length protein (human) from insect cells	1:50	ab5181, Abcam, Cambridge, UK
TRF2	Mouse monoclonal	His-tagged, fusion protein, corresponding to full-length TRF2 (Telomeric Repeat binding Factor 2)	1:100	05-521, Millipore, Temecula, CA, USA
VASA/DDX4	Mouse monoclonal	A synthetic peptide corresponding to residues near the N-terminus of human DDX4	1:50	MA5-15565, Pierce, Waltham, MA, USA
5-Methylcytosine	Mouse monoclonal	Detects methylated DNA or RNA	1:200	NA81, Calbiochem, Merck, Burlington, MA, USA

**Table 3:** The primers applied in RT-PCR for *POU5F1* and meiotic proteins.

Gene	Forward Primer Sequence	Reverse Primer Sequence	Amplicon Length	Tann (°C)	Reference	Sequence ID
*POU5F1-A (Oct4A)*	TCGCAAGCCCTCATTTCACC	GCCAGGTCCGAGGATCAAC	157	56	[109]	NM_002701.5
*POU5F1-B (Oct4B)*	AGACTATTCCTTGGGGCCACAC	GGCTGAATACCTTCCCAAATAGA	244	58	[109]	NM_203289.5
*POU5F1-B1(Oct4B1)*	TGACCGCATCTCCCCTCTAA	AGCTTACCACCTCTTCCCAG	134	58	[109]	NM_001285986.1
*MOS*	CGGTGTTCCTGTGGCCATAA	GCAGGCCGTTCACAACATC	250	58	[75]	NM_005372.1
*REC8*	TGAGGGTGAATGTGGTGAAA	CTGGGATTGCAGCCTCTAAG	400	56	[75]	NM_005132.2
*DMC1*	AGCAGCAAAGTTCCATGAAG	TGAGCTCTCCTCTTCCCTTT	300	54	[75]	NM_007068.3
*SPO11*	TGAGGTTCTTGCATCTATAGAAA	AAATTTTTGAGCTGATTTTGGTG	240	58	in house	NM_012444.2
*ACTB*	AGTGTGACGTGGACATCCG	AATCTCATCTTGTTTTCTGCGC	349	56	[110]	NA

**Table 4:** The primers used for selfie-digital PCR.

Gene Symbol	Amplicon Size	Forward Primer Sequence 5′-3′	Reverse Primer Sequence 5′-3′
*RAC-1*	75 bp.	AAACCGGTGAATCTGGGCTT	CGGATAGGATAGGGGGCGTA
*POU5F1*	97 bp.	GAGTAGTCCCTTCGCAAGCC	GAGAAGGCGAAATCCGAAGC
*CDC-42*	95 bp.	TGTTGAACCAATGCTTTCTCATGT	CTCAGGCTGGCTTGTGAAGG

## Supplementary Materials

The following figures are available online at https://www.mdpi.com/1422-0067/21/8/2779/s1, Figure S1: Amoeboid giant cells formed by DOX-treated MDA MB 231 cells grown on chamber slides on days 19-21 post-DOX-treatment; Figure S2: The increased extranuclear release of the TERT-enriched DNA in late giant cells post-DOX treatment maybe associated with unscheduled DNA synthesis.

## Abbreviations

DOX	Doxorubicin
MS	Mitotic slippage
IM	Inverted meiosis
HR	Homologous recombination
PML	Promyelocytic leukemia (protein)
DSB	Double-strand break
ALT	Alternative telomere lengthening
TMM	Telomere maintenance mechanism
EMT	Epithelial–mesenchymal transition
NT	Non-treated
FBS	Fetal bovine serum
