# Supplementary material for: “Mitotic Slippage” and Extranuclear DNA in Cancer Chemoresistance: A Focus on Telomeres
Source: Int J Mol Sci. 2020 Apr 16;21(8):2779. doi: 10.3390/ijms21082779 (PMC7215480; doi:10.3390/ijms21082779)
Supplement: Supplementary file 1 [file ijms-21-02779-s001.zip › Suppl.Fig.1.pdf]

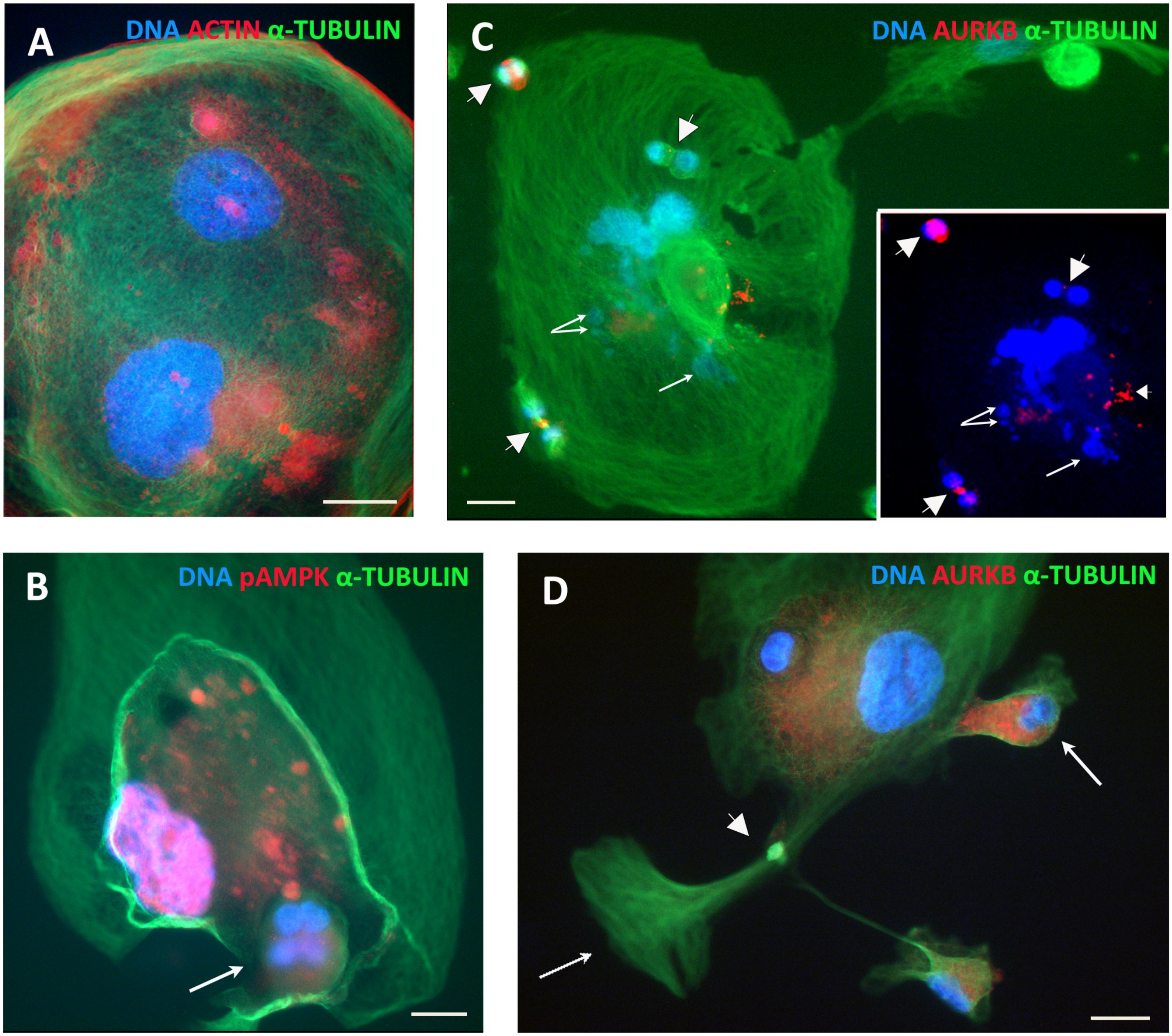

Figure S1. Amoeboid giant cells formed by DOX-treated MDA MB 231 cells grown on chamber slides on days 19-21 post-DOX-treatment. (**A**) The encysted cell, enriched with actin; (**B**) The excysting (?) tetranuclear subcell (arrow) is leaving a giant cell, while another subnucleus is positive for pAMPK inducing autophagy; (**C-D**) The features of the cooperativity between giant amoeboid cells and their progeny at late terms post- DOX-treatment. (**C**) A huge cell with the deteriorating nucleus (arrowed, better seen on insert) is homing at the same time three small cells performing mitosis (arrowheads): metaphase, anaphase, and telophase; (**D**) A giant amoeboid cell with a powerful "paddle" is budding a bi-nuclear subcell (arrow) and provides the mechanical support for the focal contact (arrowhead) of some other, small cell. Bars=25 μm.
