# Supplementary material for: “Mitotic Slippage” and Extranuclear DNA in Cancer Chemoresistance: A Focus on Telomeres
Source: Int J Mol Sci. 2020 Apr 16;21(8):2779. doi: 10.3390/ijms21082779 (PMC7215480; doi:10.3390/ijms21082779)
Supplement: Supplementary file 1 [file ijms-21-02779-s001.zip › Suppl.Fig.2.pdf]

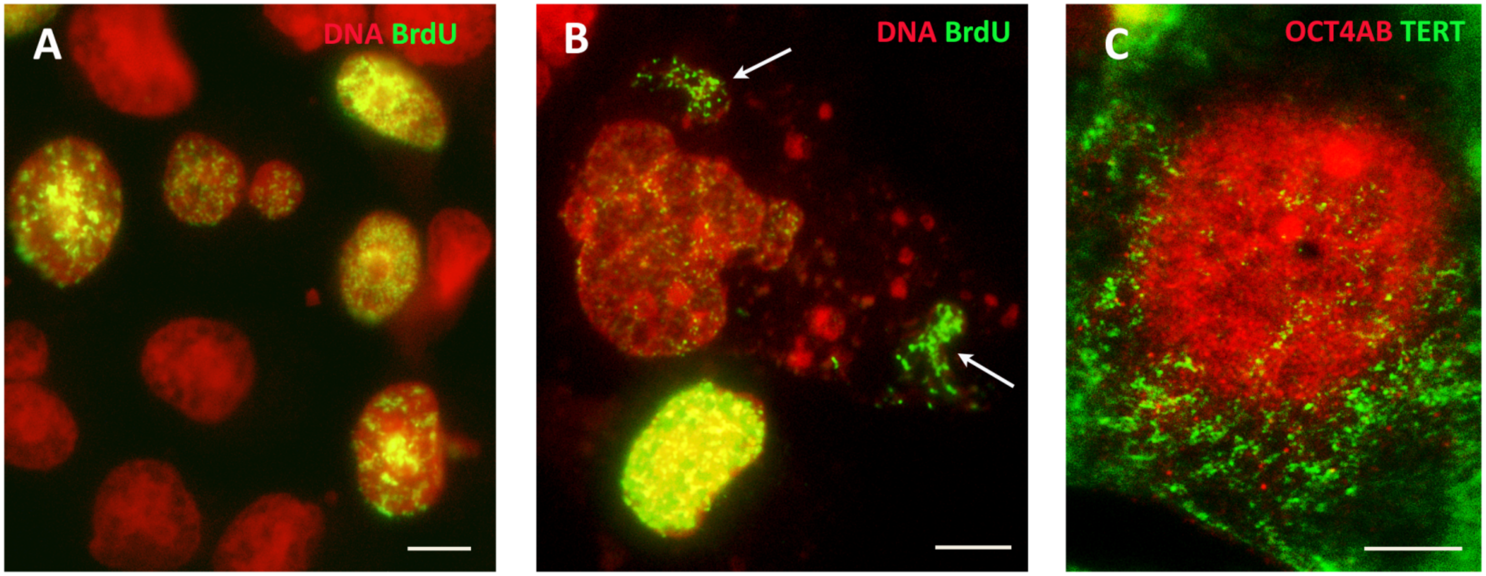

Figure S2. The increased extranuclear release of the TERT-enriched DNA in late giant cells post-DOX treatment maybe associated with unscheduled DNA synthesis. (**A , B**) - Brdu 90 min inclusion test revealed with the monoclonal antibody to BrdU, counterstained with propidium iodide. (**A**) - non-treated control: some cells are in S-phase, (**B,C**) - Day 19 post-DOX. (**B**) The scarce grains of BrdU inclusion in the giant cell nucleus and accumulation of BrdU grains in the clusters of the extranuclear DNA (arrows) suggest the release of the amplified DNA; (C) A cell with high expression of OCT4A including scarce grains of TERT in the giant cell nucleus releasing the extranuclear material enriched with TERT (double immunofluorescence, HCl pretreatment for DNA denaturation was omitted). Bars=10 μm.
